# Urinary 8-OxoGsn as a Potential Indicator of Mild Cognitive Impairment in Frail Patients With Cardiovascular Disease

**DOI:** 10.3389/fnagi.2021.672548

**Published:** 2021-08-25

**Authors:** Si-Min Yao, Pei-Pei Zheng, Wei He, Jian-Ping Cai, Hua Wang, Jie-Fu Yang

**Affiliations:** ^1^Department of Cardiology, Beijing Hospital, National Center of Gerontology, Institute of Geriatric Medicine, Chinese Academy of Medical Sciences, Beijing, China; ^2^Department of Cardiology, Peking University Fifth School of Clinical Medicine. No. 1, Beijing, China; ^3^MOH Key Laboratory of Geriatrics, Beijing Hospital, National Center of Gerontology, Beijing, China

**Keywords:** oxidative stress, 8-oxoGsn, mild cognitive impairment, frailty, cardiovascular disease

## Abstract

Oxidative RNA damage has been found to be associated with age-related diseases and 8-oxo-7,8-dihydroguanosine (8-oxoGsn) is a typical marker of oxidative modification of RNA. Urine tests are a feasible non-invasive diagnostic modality. The present study aimed to assess whether the measurement of urinary 8-oxoGsn could represent a potential early maker in mild cognitive impairment (MCI) of frail patients with cardiovascular disease (CVD). In this cross-sectional study performed in China from September 2018 to February 2019. Urinary 8-oxoGsn was measured in frail (Fried phenotype: 3–5) in patients with CVD and was adjusted by urinary creatinine (Cre) levels. Cognitive function was assessed by the Chinese version of the Mini-Mental State Examination (MMSE) and participants were classified into non-MCI (≥24) and MCI (<24) groups. Univariate and multivariate logistic regression models were used to determine the relationship between 8-oxoGsn/Cre and MCI. Receiver operating characteristic (ROC) curve analysis was used to assess the 8-oxoGsn/Cre ratio in relation to MCI in frail patients with CVD. A total of 106 elderly patients were enrolled in this study. The mean age of participants was 77.9 ± 6.8 years, the overall prevalence of MCI was 22.6% (24/106), and 57.5% (61/106) of participants were women. In the multivariate logistic regression analysis, urinary 8-oxoGsn/Cre was independently associated with MCI (odds ratio [OR] = 1.769, 95% confidence interval [CI] = 1.234–2.536, *P* = 0.002), after adjusting for age, sex, education level, marital status, and serum prealbumin levels. The area under the ROC curve was 0.786 (0.679–0.893) (*P* < 0.001), and the optimal cut-off value was 4.22 μmol/mol. The urinary 8-oxoGsn/Cre ratio showed a sensitivity of 87.5% and a specificity of 69.5%. The present study suggests the urinary 8-oxoGsn/Cre ratio may be a useful indicator for the early screening of MCI in frail patients with CVD.

**Clinical Trial Registration:**

ChiCTR1800017204; date of registration: 07/18/2018. URL: http://www.chictr.org.cn/showproj.aspx?proj=28931.

## Introduction

Mild cognitive impairment (MCI) is a transition between normal aging and dementia, and is an early indicator of dementing disorders in adults (Lovell and Markesbery, 2008). Subjects with MCI have a 10-fold increased risk of developing Alzheimer’s disease (AD) at a rate of 15% annually (Geda et al., 2013). Distinguishing between MCI individuals from individuals with no evidence of MCI (no-MCI) is an important task and requires a complete understanding of risk factors and biomarkers for early detection of MCI.

In older adults, frailty represents a state of increased vulnerability to stressor events and increases the risk of early mortality, disability, falls, and hospitalization (Fried et al., 2001). Frailty is common in patients with cardiovascular disease (CVD). However, there is a bidirectional link between frailty and CVD (Uchikado et al., 2020), whereby frailty is associated with an earlier onset of CVD, and conversely, the presence of CVD is associated with a greater incidence of frailty (Fernandes et al., 2020). These two states influence each other and worsen the prognosis of these patients. Frail patients have been associated with a significantly increased risk of developing vascular dementia, over other types of dementia, compared to non-frail participants (Robertson et al., 2013). Early detection of MCI can help to prevent vascular dementia in frail patients.

The most commonly used markers of frailty and MCI are those related to inflammatory, nutritional, vascular, and metabolic factors (Ma and Chan, 2020). In addition, frailty and cognitive decline are associated with oxidative stress, a pro-inflammatory environment leading to endothelial dysfunction, which further promotes the occurrence of the above two states (Lin and Beal, 2006; Ma and Chan, 2020).

We and other groups have found that oxidative stress contributes significantly to the pathogenesis and progression of AD (Shan and Lin, 2006; Dai et al., 2018), as well as other forms of dementia (Nunomura et al., 2004). Oxidative RNA damage can impair protein translation, and the damaged RNA can be prematurely degraded, further impairing the synthesis of essential proteins (Butterfield and Halliwell, 2019). The oxidative stress marker 8-oxo-7,8-dihydroguanosine (8-oxoGsn) can reliably be quantified in urine using an ultra-performance liquid chromatography-mass spectrometry (UPLC-MS/MS) assay and is a valid marker of RNA damage (Weimann et al., 2002). Levels of 8-oxoGsn may be associated with the pathogenesis of bipolar disorder (Knorr et al., 2019), which is a mental disorder characterized by recurrent relapses of affective episodes, cognitive impairment, and disease progression (Schneider et al., 2012). In a study of 5 patients with MCI, increased oxidative modification of RNA was found in neurons and was associated with early neurofibrillary tangles in the hippocampus/parahippocampal gyrus (Lovell and Markesbery, 2008). However, these examinations are invasive, as both require sampling of the cerebrospinal fluid and the hippocampus. In the realm of biomarker discovery, the urine is a popular matrix due to its non-invasive collection in humans and its availability in large quantities (Cook et al., 2020), it provides a precious clinical sample for early non-invasive disease diagnosis. If patients with MCI could be detected early by examination of urine, the burden on patients will be reduced.

Our previous study showed that urinary 8-oxoGsn is independently associated with frailty in elderly patients with CVD (Liang et al., 2020). However, only a few studies have investigated RNA oxidation and MCI in frail patients with CVD. The aim of this study was to assess whether 8-oxoGsn could represent a potential indicator for the MCI among frail patients with CVD.

## Materials and Methods

### Study Design and Participants

This study was a prospective cross-sectional study performed in China, which included inpatients aged ≥65 years old admitted to the Department of Cardiology from September 2018 to February 2019. Baseline assessments were carried out by experienced and trained investigators. Written informed consent was obtained from all participants. This study conformed to the Declaration of Helsinki and was approved by the Ethics Committee of Beijing Hospital (No.2018BJYYEC-121-02).

Inclusion criteria included: (1) definite diagnosis of CVD; (2) frail patients: Fried phenotype ≥3; and (3) sufficient urine samples for analysis. Exclusion criterion consisted of patients with a definite diagnosis of dementia.

Initially, 542 individuals with CVD participated in the baseline study. We excluded 348 robust individuals and 64 pre-frail participants. Of these, 24 participants without fresh urine samples were excluded, including 1 patient with AD. Finally, this study enrolled 106 participants for the determination of whether 8-oxoGsn is a useful marker for MCI in frail patients with CVD. In the supplementary materials, we also analyzed the data of 390 non-frail patients with qualified urine samples in order to establish a control group to verify the main conclusions.

### Assessment of Frailty

The Fried phenotype was used to assess frailty (Fried et al., 2001) and is based on five criteria: unintentional weight loss, self-reported exhaustion, weakness, slow walking speed, and low physical activity. Participants meeting three or more criteria were categorized as frail based on: (1) unintentional weight loss: weight decreased by >5% in the previous year; (2) self-reported exhaustion: feeling tired all of the time (at least 3 or 4 days per a week); (3) weakness: maximum grip strength of the dominant hand at ≤20% of the population distribution, adjusted for sex and body mass index; (4) slow walking speed: using the average of timed walk test over a 4-meter course, defined as walking 4-m at <0.65m/s (height ≤ 173 cm for men or ≤159 cm for women) or <0.76 m/s (height > 173 cm for men or >159 cm for women); and (5) low physical activity: <383 kcal per week for men or <270 kcal per week for women.

### Cognitive Function Assessment

Cognitive function was assessed by the Chinese version of the Mini-Mental State Examination (MMSE) (An and Liu, 2016). The MMSE tests consists of 30 items within 6 dimensions: orientation, registration, attention, language, memory, and visual construction skills (Tsoi et al., 2015). The total MMSE score ranged from 0 to 30, with higher scores reflecting better cognitive function. We treated responses of “unable to understand and answer” as “wrong” (Lv et al., 2019). Participants were classified into non-MCI (NO-MCI, ≥24) and MCI (<24) groups using the cut-off score of 24.

### Measurement of Urinary 8-OxoGsn, Creatinine and Prealbumin in All Patients

For this study, fresh midstream urine samples were obtained in the morning within 24 h after admission to hospital. All samples were coded at the moment of collection to ensure a blind study. Urine samples were stored at -80°C until they were processed. The samples were thawed at 4°C, then after centrifugation at 7500 × *g* for 5 min to remove large particles, the supernatant was used for the analysis. Urinary 8-oxoGsn levels were determined by UPLC-MS/MS, as described elsewhere (Liang et al., 2020). Creatinine (Cre) concentrations were determined in urine samples using the MicroVue Creatinine EIA kit (Hitachi Koki; Tokyo, Japan). The results for 8-oxoGsn in urine were normalized for Cre. Prealbumin was measured with immunonephelometric assays on BN II system (Siemens Healthcare; Tarrytown, New York).

### Statistical Analyses

The Kolmogorov-Smirnov test was used to verify whether continuous variables conformed to a normal distribution. Results were expressed as mean and standard deviation (normally distributed data) or median (interquartile range; non-normally distributed data). Categorical variables were expressed as numbers and percentages. The Student’s *t*-test or Mann-Whitney test for continuous data and the Fisher’s exact test or chi-square test for categorical data were used to identify statistical differences between the two groups. In our study, prealbumin was used to reflect nutritional status and high-sensitivity C-reactive protein was used to represent inflammatory state, which are commonly used markers of MCI. Univariate and multivariate logistic regression models were used to determine the relationship between the 8-oxoGsn/Cre ratio and MCI. Multivariate logistic regression was adjusted for age, sex, education level, marital status, and serum prealbumin levels (factors with a *P*-value < 0.10 in univariate analyses were entered into the multivariate model). Odds ratios (ORs) and 95% confidence intervals (CIs) were calculated in the results of the logistic regression models. Receiver operating characteristic (ROC) curve analysis was used considering the 8-oxoGsn/Cre ratio in relation to MCI in frail patients with CVD. The optimal cutoff point was calculated using the maximum value of the Youden Index (determined as sensitivity + [1-specificity]) (Schisterman et al., 2005). A *P*-value < 0.05 was considered statistically significant. All the data analyses were conducted using the IBM SPSS Statistics software program (version 24; IBM Corporation, Armonk, NY, United States). Graphs were created with GraphPad Prism version 7.0.0 for Windows (GraphPad Software San Diego, CA, United States).

## Results

**FIGURE 1:**
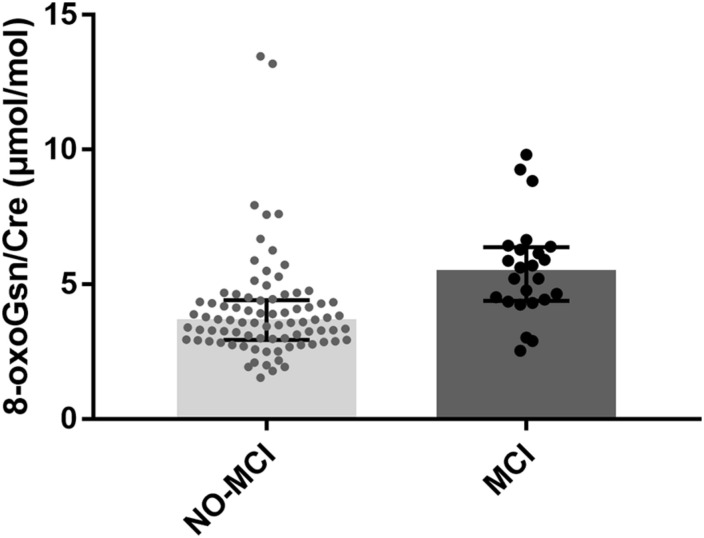
Boxplot for 8-oxoGsn/Cre in the urine samples of NO-MCI and MCI individuals. Error bars represent median with interquartile distance. 8-oxoGsn, 8-oxo-7,8-dihydroguanosine; Cre, creatinine; NO-MCI, Non-mild cognitive impairment; MCI, mild cognitive impairment.

The characteristics of the study population are presented in Table 1. Overall, a total of 106 elderly frail patients were enrolled in this study. The participants were classified into NO-MCI (*n* = 82) and MCI (*n* = 24) groups based on the MMSE scores. At baseline, the mean age of participants was 77.9 ± 6.8 years, 57.5% (61/106) of participants were women. Age (*P* < 0.001), sex (*P* = 0.003), education level (*P* < 0.001), and marital status (*P* = 0.007) showed statistically significant differences between groups. Among the five criteria of frailty, the MCI group had more patients exhibiting weakness (*P* = 0.029) compared to the NO-MCI group, no significant differences were observed regarding the proportion of individuals with unintentional weight loss (*P* = 0.183), self-reported exhaustion (*P* = 0.096), slow walking speed (*P* = 0.936), and low physical activity (*P* = 0.975). Furthermore, there were no differences in comorbidities or serum high sensitivity C-reactive protein levels among the two groups. Serum prealbumin was significantly higher in NO-MCI cases than in MCI cases (22.75 ± 5.11 vs. 19.04 ± 5.74 mg/dL; *P* = 0.003). Participants with MCI were more likely to have higher levels of urinary 8-oxoGsn/Cre [5.43 (4.39–6.38) vs. 3.60 (2.94–4.42) μmol/mol; *P* < 0.001] (Figure 1).

**TABLE 1:** The baseline characteristics of study participants by cognitive function status.

	Overall (*n* = 106)	NO-MCI (*n* = 82)	MCI (*n* = 24)	*P*
Age, year	77.9 ± 6.8	76.7 ± 6.8	82.2 ± 5.2	<0.001
Sex, female (%)	61 (57.5)	41 (50.0)	20 (83.3)	0.003
Education level, year	10 (9–15)	12 (9–15)	6 (1–11)	<0.001
Married (%)	83 (78.3)	69 (84.1)	14 (58.3)	0.007
MMSE	28 (24–29)	28 (27–29)	20 (13–23)	<0.001
Unintentional weight loss (%)	34 (32.1)	29 (35.4)	5 (20.8)	0.183
Self-reported exhaustion (%)	94 (88.7)	75 (91.5)	19 (79.2)	0.096
Weakness (%)	85 (80.2)	62 (75.6)	23 (95.8)	0.029
Slow walking speed (%)	88 (83.0)	68 (82.9)	20 (83.3)	0.963
Low physical activity (%)	97 (91.5)	75 (91.5)	22 (91.7)	0.975
Prealbumin, mg/dL	21.90 ± 5.46	22.75 ± 5.11	19.04 ± 5.74	0.003
High sensitivity C-reactive protein, mg/L	1.28 (0.58–4.68)	1.14 (0.54–4.50)	1.70 (0.59–11.23)	0.269
Coronary artery disease (%)	59 (55.7)	45 (54.9)	14 (58.3)	0.767
Hypertension (%)	79 (74.5)	60 (73.2)	19 (79.2)	0.558
Heart failure (%)	22 (20.8)	16 (19.5)	6 (25.0)	0.564
Atrial fibrillation (%)	36 (34.0)	26 (31.7)	10 (41.7)	0.370
Diabetes (%)	45 (42.5)	38 (46.3)	17 (70.8)	0.137
Previous stroke (%)	25 (23.6)	16 (19.5)	9 (37.5)	0.069
Obesity (%)	23 (21.7)	19 (23.2)	4 (16.7)	0.501

*NO-MCI, Non-mild cognitive impairment; MCI, mild cognitive impairment; MMSE, Mini-Mental State Examination; Cre, creatinine. Values are shown as mean ± standard deviation or median (interquartile range) or n (%).*

Three hundred and ninety non-frail patients with CVD were divided into two groups according to the MMSE. The characteristics of non-frail patients are shown in Supplementary Table 1. There was no difference in levels of urinary 8-oxoGsn/Cre between the two groups [NO-MCI: 3.20(2.48–4.14) μmol/mol vs. MCI: 3.69(3.10–4.54) μmol/mol, *P* = 0.186].

Univariate analysis demonstrated that age (*P* = 0.001), sex (*P* = 0.006), education level (*P* < 0.001), marital status (*P* = 0.009), serum prealbumin (*P* = 0.005), and urinary 8-oxoGsn/Cre (*P* = 0.005) were associated with MCI. To better explore the association between 8-oxoGsn/Cre and MCI, a multivariate logistic regression model was built. The urinary 8-oxoGsn/Cre ratio was independently associated with MCI (OR = 1.769, 95% CI = 1.234–2.536, *P* = 0.002), after adjusting for age, sex, education level, marital status, and serum prealbumin. Age (OR = 1.202, 95% CI = 1.045–1.383, *P* = 0.010) and education level (OR = 0.742, 95%CI = 0.621–0.886, *P* = 0.001) were independently associated with MCI in frail patients with CVD (Table 2). In non-frail patients, univariate logistic regression analysis demonstrated that urinary 8-oxoGsn/Cre was not associated with MCI in non-frail patients (OR = 1.152, 95%CI = 0.933–1.423, *P* = 0.189).

**TABLE 2:** Univariate and multivariate logistic regression.

Variables	Univariate analysis				Multivariate analysis			
	OR	95% CI		*P*	OR	95% CI		*P*
		Lower	Upper			Lower	Upper	
Age, year	1.144	1.056	1.240	0.001	1.202	1.045	1.383	0.010
Female	5.000	1.570	15.910	0.006	0.290	0.059	1.424	0.127
Higher level of education, year	0.724	0.626	0.836	<0.001	0.742	0.621	0.886	0.001
Married	0.264	0.097	0.720	0.009	1.013	0.194	5.281	0.988
Unintentional weight loss	0.481	0.163	1.422	0.186	-	-	-	-
Self-reported exhaustion	1.027	0.199	5.302	0.975	-	-	-	-
Weakness	7.419	0.941	58.480	0.057	-	-	-	-
Slow walking speed	1.029	0.305	3.480	0.963	-	-	-	-
Low physical activity	0.355	0.101	1.242	0.105	-	-	-	-
8-oxoGsn/Cre, μmol/mol	1.415	1.112	1.800	0.005	1.769	1.234	2.536	0.002
Prealbumin, mg/dL	0.876	0.798	0.962	0.005	0.904	0.793	1.031	0.133
High sensitivity C-reactive protein, mg/L	1.018	0.979	1.059	0.362	-	-	-	-
Coronary artery disease	1.151	0.458	2.891	0.765	-	-	-	-
Hypertension	1.393	0.464	4.184	0.554	-	-	-	-
Heart failure	1.375	0.470	4.022	0.561	-	-	-	-
Atrial fibrillation	1.538	0.604	3.920	0.367	-	-	-	-
Diabetes	0.477	0.179	1.272	0.139	-	-	-	-
Previous stroke	2.475	0.919	6.665	0.073	-	-	-	-
Obesity	0.663	0.202	2.179	0.499	-	-	-	-

*OR, odds ratio; CI, confidence interval; 8-oxoGsn, 8-oxo-7,8-dihydroguanosine; Cre, creatinine.*

**FIGURE 2:**
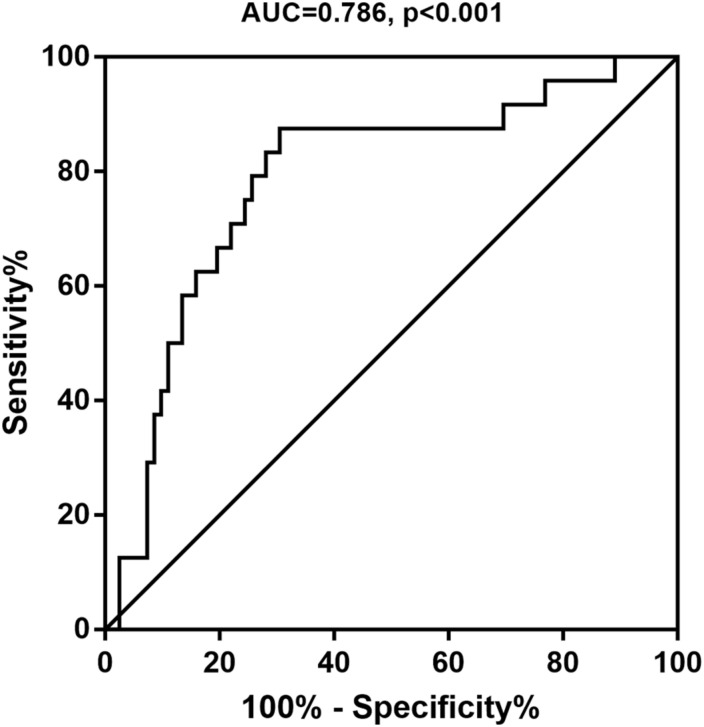
Receiver operating characteristic curve for the 8-oxoGsn/Cre to predict MCI. 8-oxoGsn, 8-oxo-7,8-dihydroguanosine; Cre, creatinine; MCI, mild cognitive impairment; AUC, areas under the curve.

A ROC curve analysis was performed to estimate the diagnostic potential of urinary the 8-oxoGsn/Cre ratio for MCI. The area under the ROC curve (AUC) was 0.786 (0.679–0.893) (*P* < 0.001) (Figure 2). The optimal cut-off value of the urinary 8-oxoGsn/Cre ratio by the maximal Youden index was 4.22 μmol/mol. It showed a sensitivity of 87.5% and a specificity of 69.5%. Its positive predictive and negative predictive values were 74.2% and 84.8%, respectively, and its positive likelihood ratio and negative likelihood ratio were 2.87 and 0.18.

## Discussion

In this cross-sectional study, we reported the overall prevalence of MCI among frail patients with CVD was 22.6%, which was similar to previous findings (Petersen et al., 2010). We confirmed that urinary 8-oxoGsn/Cre ratios were significantly higher in MCI patients compared to patients with no evidence of MCI. We determined that urinary 8-oxoGsn/Cre could independently and effectively evaluate MCI in frail patients with CVD.

The oxidative modifications of RNA can be measured by urinary 8-oxoGsn levels (Larsen et al., 2019), which is the focus of the present study. The use of feasible and reliable biomarkers to identify patients with MCI is a challenge that needs to be addressed. Such indicators would provide a more accurate detection of dementia in early disease stages, when cognitive decline can still be potentially reverted. Individuals with MCI have shown alterations in the antioxidant system, which is designed to counteract the potentially hazardous reactions initiated by oxidative stress (Stephan et al., 2012). Nucleic acids are constantly oxidized within the cell and nuclear DNA is double stranded and is complexed with protective proteins. Pena-Bautista et al. showed that the DNA oxidation marker 8-hydroxy-2′-deoxyguanosine was able to distinguish between AD and healthy participants (Pena-Bautista et al., 2019). However, RNA is more vulnerable to oxidative stress than DNA because it is single-stranded and lacks protective histones (Liu et al., 2016). RNA damage is a valid marker that may provide useful information for early identification of MCI.

Accurate and early detection of oxidative modification markers indicative of chronic disease progression can provide useful diagnostic information. MCI reflects the transition between normal aging and dementia, and is the earliest clinical manifestation of AD. Perez et al. found that titanium dioxide nanoparticles induced strong oxidative stress in astrocytes, cells that play key roles in neuronal homeostasis and their dysfunction can lead to MCI (Perez-Arizti et al., 2020). Keller et al. showed significantly increased protein carbonyl formation and increased levels of lipid peroxidation in the temporal lobe of MCI subjects compared to healthy subjects (Keller et al., 2005). Ding et al. showed significantly elevated 8-hydroxyguanine immunoreactivity in the inferior parietal lobule of subjects early in disease progression (Ding et al., 2006).

Our findings suggest that an increase in the urinary 8-oxoGsn/Cre ratio may be a useful indicator for the early screening of MCI in frail patients with CVD. Previous studies on oxidative stress and cognitive impairment have mainly focused on brain tissue and cerebrospinal fluid rather than on urine samples. Nunomura et al. suggested that RNA oxidation is a prominent feature of neuronal vulnerability in patients with AD (Nunomura et al., 1999) and dementia (Nunomura et al., 2002). Our previous study found that the presence of large amounts of 8-oxoGsn in the RNA could promote the secretion of pathogenic amyloid-β peptides *in vivo* (Dai et al., 2018), and this mechanism could contribute to the accumulation of amyloid-β plaques as is observed in the brains of AD patients. Lovell et al. described the presence of increased RNA oxidative modifications in neurons undergoing early neurofibrillary tangle formation in the hippocampus/parahippocampal gyrus of MCI and late-stage AD subjects (Lovell and Markesbery, 2008). Further, the levels of RNA oxidative damage observed in MCI were comparable to those observed in late-stage AD (Lovell and Markesbery, 2008).

The usual risk factors associated with conversion of individuals from cognitively normal status into dementia and AD are also possible risk factors for transitions into MCI (Kryscio et al., 2006). Our findings showing that age and education levels were independently associated with MCI are consistent with the current literature (O’Bryant et al., 2013; Wong et al., 2019). It is estimated that between 10 and 30% of all adults aged 65 and above experience MCI (Geda et al., 2013). In a longitudinal study at the University of Kentucky AD Center, it was shown that age affected the ORs of individuals transitioning to MCI as well as that of onset of dementia or death (Kryscio et al., 2006). In an analysis of six international longitudinal studies, a higher education level was associated with a lower risk of transitioning from MCI in individuals with no prior evidence of MCI. Furthermore, those with a higher level of education and socioeconomic status experienced longer non-impaired life expectancies (Robitaille et al., 2018).

Cardiovascular risk factors and diseases are recognized as predictors of age-related cognitive decline and dementia (Sahathevan et al., 2012). MCI is an important under-researched complication of stroke and transient ischemic attack (Drozdowska et al., 2020). However, it is important to note that no clear correlation between cardiovascular risk factors or diseases and MCI was identified in our study. Nonetheless, these findings were not consistent with our hypothesis. One possible reason for this inconsistency may be that we excluded patients with dementia from our study, and only patients with mild cognitive decline were enrolled, and thus there was an insufficient number of cases with CVD to detect early changes in cognitive function. Second, previous studies have found that some forms of CVD and risk factors, such as heart failure (Yao et al., 2020), coronary artery disease (Ma et al., 2020), stroke (Chumha et al., 2020), atrial fibrillation (Guo et al., 2020), and obesity (Chan et al., 2020), are significantly associated with frailty, thus the relationship between CVDs or risk factors and MCI may be weakened in frail patients. Further research on CVDs and MCI in frail patients should be performed in the future.

The strengths of the current study include the analysis of oxidized nucleosides using UPLC-MS/MS, which is considered the reference standard method due to high specificity toward the RNA forms.

Our study has several limitations. First, the small sample size hampers the generalizability of our findings. Second, our cross-sectional study did not allow to draw any causative conclusions, nor could it identify risk factors associated with MCI. A long-term follow-up study in this population is currently being conducted by our group, which will verify whether these patients develop dementia in the future. Third, we studied the frail patients with CVD, which is a specific part of the population, and the results were not validated in the general population. Lastly, MMSE is a screening tool to identify MCI but it is not a diagnostic tool; thus, we failed to conduct a detailed subgroup analysis of patients with stratified by cognitive level. Nonetheless, the MMSE is the most widely used tool for evaluating MCI and is supported by a high degree of popularization and application.

In conclusion, the present study suggests the urinary 8-oxoGsn/Cre ratio may be a useful indicator for the early screening of MCI in frail patients with CVD. This indicator will enhance our understanding of the underlying pathological processes causative of MCI and the potential risk factors for early dementia progression. With the aging population, the number of frail patients with CVD may continue to increase. Early recognition of cognitive dysfunction and early intervention may help to improve the quality of life and prognosis of these patients.

## Data Availability Statement

The original contributions presented in the study are included in the article/Supplementary Material, further inquiries can be directed to the corresponding authors.

## Ethics Statement

The study involving human participants was reviewed and approved by the Ethics Committee of Beijing Hospital, China (ID number: 2018BJYYEC-121-02), the version date of the protocol approved by ethics is September 18, 2018, and the version number is 1.0. The patients/participants provided their written informed consent to participate in this study.

## Author Contributions

HW, J-FY, and J-PC designed the research. S-MY and P-PZ contributed to the development of the conceptualization and methodology and wrote the manuscript. WH and S-MY analyzed data. All of the authors read the draft, made contributions, and approved the final manuscript.

## Conflict of Interest

The authors declare that the research was conducted in the absence of any commercial or financial relationships that could be construed as a potential conflict of interest.

## Publisher’s Note

All claims expressed in this article are solely those of the authors and do not necessarily represent those of their affiliated organizations, or those of the publisher, the editors and the reviewers. Any product that may be evaluated in this article, or claim that may be made by its manufacturer, is not guaranteed or endorsed by the publisher.

## Abbreviations

MCI: Mild cognitive impairment
AD: Alzheimer’s disease
CVD: Cardiovascular disease
8-oxoGsn: 8-oxo-7,8-dihydroguanosine
MMSE: Mini-Mental State Examination
UPLC-MS/MS: Ultra-performance liquid chromatography-mass spectrometry
NO-MCI: Non-mild cognitive impairment
Cre: Creatinine
OR: Odds ratio
CI: Confidence interval
ROC: Receiver operating characteristic curve
AUC: Area under the ROC curve.
