# Supplementary material for: Urinary 8-OxoGsn as a Potential Indicator of Mild Cognitive Impairment in Frail Patients With Cardiovascular Disease
Source: Front Aging Neurosci. 2021 Aug 25;13:672548. doi: 10.3389/fnagi.2021.672548 (PMC8439254; doi:10.3389/fnagi.2021.672548)
Supplement: Supplementary file 1 [file Table_1.DOC]

Supplementary Table S1. The baseline characteristics of non-frail patients by cognitive function status.

|  | Overall (n=390) | NO-MCI (n=357) | MCI (n=33) | P |
| --- | --- | --- | --- | --- |
| Age, year | 74.1±6.0 | 73.5±5.8 | 80.4±5.6 | ＜0.001 |
| Sex, female (%) | 183(46.9) | 167(46.8) | 16(48.5) | 0.851 |
| Education level, year | 12(8-16) | 12(9-15) | 6(3-9) | ＜0.001 |
| Married (%) | 330(84.6) | 308(86.3) | 22(66.7) | 0.003 |
| MMSE | 27(24-30) | 28(27-30) | 21(17-22) | ＜0.001 |
| Unintentional weight loss (%) | 25(6.4) | 22(6.2) | 3(9.1) | 0.511 |
| Self-reported exhaustion (%) | 150(38.5) | 142(39.8) | 8(24.2) | 0.079 |
| Weakness (%) | 171(44.3) | 147(41.6) | 24(72.7) | 0.001 |
| Slow walking speed (%) | 258(69.2) | 245(70.8) | 13(48.1) | 0.014 |
| Low physical activity (%) | 207(53.1) | 193(54.1) | 14(42.4) | 0.200 |
| Prealbumin, mg/dL | 25.18±5.78 | 25.37±5.79 | 22.80±5.20 | 0.019 |
| High sensitivity C-reactive protein, mg/L | 0.96(0.56-1.95) | 0.94(0.56-1.92) | 1.41(0.63-2.57) | 0.535 |
| Coronary artery disease (%) | 220(56.4) | 199(55.7) | 21(63.6) | 0.382 |
| Hypertension (%) | 283(72.9) | 258(72.5) | 25(78.1) | 0.491 |
| Heart failure (%) | 35(9.0) | 23(6.5) | 12(37.5) | ＜0.001 |
| Atrial fibrillation (%) | 74(19.1) | 62(17.4) | 12(37.5) | 0.006 |
| Diabetes (%) | 122(31.4) | 111(31.2) | 11(34.4) | 0.709 |
| Previous stroke (%) | 56(14.4) | 45(12.6) | 11(34.4) | 0.001 |
| Obesity (%) | 76(19.5) | 68(19.0) | 8(24.2) | 0.471 |
| 8-oxoGsn/Cre (μmol/mol) | 3.25(2.52-4.11) | 3.20(2.48-4.14) | 3.69(3.10-4.54) | 0.186 |
